# Kikuchi-Fujimoto disease: three case reports

**DOI:** 10.1590/S1516-31802010000400011

**Published:** 2010-07-01

**Authors:** Alexandre de Andrade Sousa, João Marcos Arantes Soares, Marco Homero de Sá Santos, Marcelo Portes Rocha Martins, José Maria Porcaro Salles

**Affiliations:** I MD. Surgeon in the Head and Neck Group, Instituto Alfa de Gastroenterologia (IAG), Hospital das Clínicas, Universidade Federal de Minas Gerais (UFMG), Belo Horizonte, Minas Gerais, Brazil.; II PhD. Surgeon in the Head and Neck Group, Instituto Alfa de Gastroenterologia (IAG), Hospital das Clínicas, Universidade Federal de Minas Gerais (UFMG), Belo Horizonte, Minas Gerais, Brazil.; III MD. Resident in Head and Neck Surgery, Hospital das Clínicas, Universidade Federal de Minas Gerais (UFMG), Belo Horizonte, Minas Gerais, Brazil.; IV MD. Resident in General Surgery, Hospital das Clínicas, Universidade Federal de Minas Gerais (UFMG), Belo Horizonte, Minas Gerais, Brazil.; V MD. Professor of Head and Neck Surgery, Universidade Federal de Minas Gerais (UFMG), and Head of the Head and Neck Group, Instituto Alfa de Gastroenterologia (IAG), Hospital das Clínicas, Universidade Federal de Minas Gerais (UFMG), Belo Horizonte, Minas Gerais, Brazil.

**Keywords:** Kikuchi disease, Histiocytic necrotizing lymphadenitis, Lymphatic diseases, Neck, Lymph nodes, Doença de Kikuchi, Linfadenite histiocítica necrosante, Doenças linfáticas, Pescoço, Linfonodos

## Abstract

**CONTEXT:**

Kikuchi-Fujimoto disease (KFD) manifests in most cases as unilateral cervical lymphadenomegaly, with or without accompanying fever. The disease mainly affects young women and has a self-limited course. It is more common in oriental countries, with few reports of its occurrence in Brazil. KFD should be included in the differential diagnosis of suspected cases of viral infections, tuberculosis, reactive lymphadenitis, systemic lupus erythematosus and metastatic diseases. It can be histologically confused with lymphoma. The disease is benign and self-limiting and an excisional biopsy of an affected lymph node is necessary for diagnosis. There is no specific therapy.

**CASE REPORTS:**

This study reports on three cases of non-Asian female patients with KFD who were attended at our service between 2003 and 2006. A review of the literature was carried out, with a systematic search on this topic, with the aim of informing physicians about this entity that is manifested by cervical masses and fever.

## RESUMO

**CONTEXTO:**

A doença de Kikuchi-Fujimoto (DKF) se manifesta na maioria das vezes como linfadenomegalia cervical unilateral, que pode ser acompanhada de febre. Afeta principalmente mulheres jovens, com evolução autolimitada. A prevalência desta doença é mais comum no oriente, havendo poucos relatos de sua ocorrência em nosso meio. DKF deve ser incluída no diagnóstico diferencial de casos suspeitos de infecções por vírus, tuberculose, linfadenite reacional, lúpus eritematoso sistêmico e doença metastática. Histologicamente pode ser confundida com linfoma. A doença é de caráter benigno, auto-limitada, a biópsia excisional do linfonodo acometido é necessária para o diagnóstico e não há tratamento específico.

**RELATO DE CASOS:**

Este trabalho relata três casos de pacientes não asiáticas do sexo feminino com DKF, atendidas em nosso serviço entre 2003 e 2006, e faz revisão da literatura com uma busca sistematizada sobre o assunto, com o objetivo de informar os médicos sobre essa entidade que se manifesta com massa cervical e febre.

## INTRODUCTION

Cervical lymphadenomegaly is a frequent clinical sign and may correspond to a series of diseases that require diverse work-ups and therapies.

Kikuchi-Fujimoto disease (KFD) is a form of histiocytic necrotizing lymphadenitis first described in Japan in 1972, almost simultaneously by Kikuchi and Fujimoto.^1-3^ They studied patients treated for lymphoma who evolved surprisingly well, showing much faster recovery than expected. In fact, these patients did not have lymphoma but a condition that has been called Kikuchi-Fujimoto disease since its initial description.^4^

KFD mainly affects young women of Asian origin (the female-to-male ratio of occurrence is 4:1).^1,5,6^ KFD should be included in the differential diagnosis of suspected cases of viral infections, tuberculosis, reactive lymphadenitis, systemic lupus erythematosus (SLE) and metastatic diseases. It can be histologically confused with lymphoma.

Clinically, KFD manifests as cervical (usually unilateral) lymphadenopathy with fever and is frequently associated with other nonspecific symptoms.^5,7^ Laboratory test results are almost always unchanged, except for erythrocyte sedimentation rate and C-reactive protein levels.^3,7^ An excisional biopsy from an affected lymph node is necessary for diagnosis.^5^ The disease is benign and self-limiting.^3,6,7^ Rheumatological diseases such as SLE and Sjögren’s syndrome may be associated with KFD, and therefore, investigation of the presence of these diseases and patient follow-up are necessary.^8^

The etiology of Kikuchi-Fujimoto disease is uncertain, and speculations exist regarding relationships with previous viral infections or autoimmune processes.^1-3^

We present three cases involving female patients of non-Asian origin with Kikuchi-Fujimoto disease who were seen between 2003 and 2006 at our outpatient department, along with a short review of the literature.

## CASE REPORTS

### Case 1

A 24-year-old non-Asian white woman came to the outpatient department with complaints of a cervical mass on the left side of her neck, which had existed for a month. The mass showed fast growth and was mildly painful. The patient did not report any other complaints.

Oroscopy and indirect laryngoscopy did not reveal any abnormalities. A hard, semi-fixed lymph node mass adhering to the sternocleidomastoid muscle was detected in the left cervical area, at levels II and III, and other lymph nodes were detected at levels IV and V. No lymph nodes were palpable on the right side of the neck. Serological tests for infectious diseases, a purified protein derivative tuberculosis test, blood counts and a chest x-ray were performed. All the tests were normal. Fine-needle aspiration biopsy revealed nonspecific lymphadenitis associated with the presence of numerous mature lymphocytes and no signs of malignancy (although lymphoma could not be ruled out). Cervical computed tomography showed the presence of a left cervical mass (cervical lymphadenomegaly, coalescent, at levels II, III, IV and V). An excisional biopsy was performed on the lymph node, and the anatomopathological diagnosis determined was chronic necrotizing lymphadenitis, or Kikuchi-Fujimoto disease.

When asked, the patient recalled that she had had flu-like symptoms accompanied by high fever a few days before the onset of the current symptoms. The patient was followed up on an outpatient basis after associated SLE had been ruled out.

### Case 2

A 14-year-old non-Asian white girl sought medical care with complaints of a left supraclavicular mass. Palpation revealed cervical lymphadenomegaly of fibroelastic consistency in the left posterior triangle of the neck (level V). The remaining physical examinations were normal, and oroscopy and indirect laryngoscopy did not reveal any abnormalities. The patient did not have any other associated complaints.

An excisional biopsy was performed on one of the lymph nodes, and histopathological analysis revealed this to be a case of histiocytic necrotizing lymphadenitis (Kikuchi-Fujimoto disease).

The patient progressed well but presented with high fever on the fifth postoperative day. This was not correlated with any infectious symptoms and regressed spontaneously. Remission from the cervical lymphadenomegaly was achieved slowly after the fever. Associated rheumatological diseases were ruled out.

### Case 3

A 34-year-old non-Asian white woman sought medical care because of a two-month history of cervical lymphadenomegaly, associated with eight days of fever at the time of the occurrence of the nodules. Thyroid ultrasound and serological tests were normal. Otorhinolaryngological examination did not show any abnormalities. An excisional biopsy was performed on one of the lymph nodes and histopathological analysis revealed signs and symptoms of necrotizing histiocytic lymphadenitis corresponding to Kikuchi-Fujimoto disease.

The patient was followed up on an outpatient basis and showed spontaneous remission of lymphadenomegaly.

## DISCUSSION

KFD is rare in Brazil, with few reports in the literature.^5^ This disorder is therefore frequently underdiagnosed. Many cases are given a presumptive diagnosis of viral infections, especially among young patients with little swelling of the lymph nodes or pain. KFD has no pathognomonic clinical signs or symptoms, and a definitive diagnosis can only be made histologically using lymph node biopsy tissue.^1,4^

KFD is known to be a disease without a specific, confirmed etiology. Several infectious agents (Epstein-Barr virus, human herpesvirus, HIV, HTLV1, dengue virus, parvovirus B19, *Yersinia enterocolitica*, *Bartonella*, *Brucella* and *Toxoplasma*) have been suggested as possible etiological agents, but none have been confirmed. The histopathological features of these lymphadenitis diseases differ from those of KFD. Some authors have hypothesized a possible link between SLE and KFD. No studies have confirmed any positive serological findings relating to antinuclear antibodies, rheumatoid factors or other immunological parameters, as signs of the autoimmune nature of this disease.^1-3,9-11^ Recent studies have demonstrated activated CD8+ cells after viral infection in lymph nodes, which may induce apoptosis of CD4+ lymphocytes. Hypothetically, apoptotic lymphocytes could deliver nuclear antigens and trigger autoimmune T and B cells to produce antinuclear antibodies.^12,13^

The main clinical manifestation of KFD is lymphadenomegaly, which is cervical in 70% to 98% of cases and generally occurs in the jugular lymph nodes and posterior cervical chain.^7,11^ However, the axillary, intraparotid, mesenteric, thoracic and inguinal chains may also be involved.^6,7^ The lymph nodes are mainly small (less than 3 cm), mobile and painless.^3,6,7^ Fever (usually high) is the first symptom in 30% to 50% of the cases; weight loss is observed in 10% and shivering in 4%.^7,9,14,15^

Other less common manifestations include skin rash, gastrointestinal abnormalities and night sweating.^7^ Skin lesions, which are observed in about 30% of patients, are nonspecific and include erythematous papules, plaques, acneiform or morbilliform lesions and facial erythema, in addition to possible mucosal involvement.^5^ Skin biopsies usually show lymphohistiocytic infiltrates accompanied by non-neutrophilic karyorrhectic debris, similar to what is observed in the lymph nodes that are involved.^2,9^

The results from a wide range of laboratory tests are usually normal in patients with KFD. Approximately 25% to 50% of the patients present with leucopenia, frequently accompanied by lymphocytosis and atypical lymphocytes. The erythrocyte sedimentation rate is higher than 60 mm/h in 70% of the patients.^1,3,7,9,16^

Computed tomography and magnetic resonance imaging do not yield features that distinguish KFD from other diseases that commonly involve lymph nodes, such as lymphoma, tumor metastases or tuberculosis.^1^

The diagnosis of KFD is based on histopathological analyses that show occurrences of confluent necrotic areas surrounded by clusters of small lymphocytes, histiocytes, immunoblasts and plasma cells in the absence of neutrophils.^5^ Immunohistochemistry using the CD68 marker shows the presence of immunoblasts and cell infiltrates, and this finding is suggestive of skin involvement. The lack of detection of monoclonal lymphocyte receptors rules out the possibility of lymphoma.^8,11,17,18^ Fine-needle aspiration biopsy has an accuracy of 56%.^1,3,8,19^

The differential diagnosis includes lymphoma, SLE, cat-scratch disease, Still’s disease, infectious mononucleosis, drug eruptions,^5^ viral lymphadenitis and tuberculosis.^5,7,10,12,17,18^

No therapy has been found to be effective so far for the treatment of histiocytic necrotizing lymphadenitis. Spontaneous regression is generally observed, and this occurs within one to six months. Some studies have suggested that there might be faster regression of the symptoms after administration of prednisolone, ciprofloxacin and minocycline, but currently, no consensus exists.^3,6,7^ A low recurrence rate has been described in 3% to 4% of the cases, and recurrence may occur as much as eight to nine years later.^1,5^

There are reports of associations between SLE and KFD. Such associations are not well defined, but some authors have recommended following up patients clinically for evidence of autoimmune disease.^9,14,18^ Yilmaz et al. believed that SLE and KFD might share a common hyperimmune reaction directed against different antigenic stimuli but that they then diverge into two separate manifestations: one compromising lymph nodes with a characteristic pattern of reactivity, named KFD, and the other manifesting itself as SLE.^10^

After a systematic search in the main data bases, using the MeSH terms "Histiocytic", "Necrotizing" and "Lymphadenitis", from 1999 to 2009 (Table 1), we identified some related studies. We observed that the case series came from Asian countries, while the case reports usually came from Western countries. These describe diagnostic difficulties and clinical, imaging and laboratory findings, along with makings comments regarding therapy and follow-up. As in the present paper, other authors have reported a lack of knowledge regarding this disease, along with lack of personal experience of case follow-up.

**Table 1.** Systematic search in the main databases, using the MeSH terms "Histiocytic", "Necrotizing" and "Lymphadenitis", from 1999 to 2009

Database	Search strategy	Results		
Cochrane Library	Histiocytic (MeSH) Necrotizing (MeSH) Lymphadenitis (MeSH)	0 papers		
Lilacs	Linfadenite histiocitica necrosante (Subject description)	18 found	18 related	15 case reports 7 narrative reviews 3 letters to editor
PubMed	Histiocytic (MeSH) Necrotizing (MeSH) Lymphadenitis (MeSH)	317 found	222 related	163 case reports 30 case series 30 pathogenesis studies 6 differential diagnosis studies 67 narrative reviews 11 diagnosis studies 1 case control study

Lilacs = Literatura Latino-Americana e do Caribe em Ciências da Saúde; MeSH = Medical Subject Headings; PubMed = United States National Library of Medicine and National Institutes of Health.

The few studies^2,3,16^ that have attempted to describe the pathogenesis of KFD, with hypotheses regarding its origin, the evolution of the disease and associations with other pathological conditions, have been from Asian countries. It appears that the numbers of patients with KFD in that region are sufficient for such studies. The present review illustrates the need for consistent molecular and serological analysis in order to clarify the etiology and pathogenesis.

In the Latin American literature, the published papers include case reports or case series (Table 1). So far, we have found 26 reported cases of KFD, most in female patients (between 12 and 58 years of age), which is consistent with our case series. Of the 26 cases, only four occurred in men. There was a high association with systemic lupus erythematosus (eight cases). The clinical presentation was cervical lymphadenomegaly in almost all the cases, although one presented mediastinal lymphadenomegaly. The diagnosis was concluded through histological evaluations on diseased lymph nodes, although the initial diagnostic hypotheses suggested lymphoma, tuberculosis, Wegener granulomatosis, and other infectious diseases.

There are no reports in which the initial diagnostic hypothesis was primarily KFD. In other words, KFD was not considered to be a diagnostic possibility prior to biopsy. Treatment was symptomatic in most cases, with spontaneous remission being the rule, except for cases associated with lupus, in which steroid treatment was needed to control the rheumatic disease. The reported prognosis for these patients was good, with exception of one patient who was diagnosed through necropsy; however, this patient had KFD in association with lupus.^20-22^

## CONCLUSION

In conclusion, once a diagnosis of Kikuchi-Fujimoto disease has been made, it is the physicians’ role to reassure and inform patients about the self-limited course of the clinical symptoms and to follow patients up on an outpatient basis because of the possible association of this disease with rheumatological diseases.
